# Epigenetic Modulation by Luteolin, Quercetin and Apigenin in Systemic Lupus Erythematosus and Rheumatoid Arthritis: Mechanisms and Therapeutic Perspectives

**DOI:** 10.3390/ijms27167365

**Published:** 2026-08-18

**Authors:** Cletus Ekwosi, Anna Kasprzyk-Pawelec, Monika Janczarek, Maciej Masłyk

**Affiliations:** 1 Department of Molecular Biology, Institute of Biological Sciences, The John Paul II Catholic University of Lublin, Konstantynów 1i St., 20-708 Lublin, Poland; cletus.ekwosi@shanahanef.org; 2 Department of Industrial and Environmental Microbiology, Institute of Biological Sciences, Maria Curie-Skłodowska University, Akademicka 19 St., 20-033 Lublin, Poland; monika.janczarek@mail.umcs.pl; 3 Department of Biochemistry and Molecular & Cellular Biology, Georgetown University Medical Center, Washington, DC 20007, USA; ak1801@georgetown.edu

**Keywords:** flavonoids, systemic lupus erythematosus, rheumatoid arthritis, epigenetic DNA, histone modifications, DNMTs, HDACs, miRNAs

## Abstract

For many years, plants have long served as sources of potential therapeutic agents. Recently, there has been a growing interest in investigating the effects of plant bio-active compounds on epigenetic modulation of several processes—among them, modifications of DNA, histones, and microRNAs that influence gene expression. Aberrant epigenetic changes can affect key cellular processes by altering the expression of many important genes, leading to disturbances in human cells, and consequently, to various diseases including systemic autoimmune diseases. However, these modifications are reversible, offering a unique way for therapeutic intervention. Many plant-derived compounds possess the potential to modify these abnormal epigenetic modifications. Among these are apigenin, quercetin and luteolin, which can alter the level of histone acetylation, DNA methylation and miRs in cells. This review highlights these abnormal epigenetic alterations in two important autoimmune diseases in humans, i.e., lupus erythematosus and rheumatoid arthritis, and discusses the promising efficacy of these bio-active compounds in mitigating these deleterious epigenetic signatures. Furthermore, this review addresses ongoing clinical investigations to evaluate the therapeutic potential of these plant-derived compounds in the treatment of both systemic lupus erythematosus and rheumatoid arthritis, along with their limitations and challenges.

## 1. Introduction

Autoimmune diseases are chronic, perplexing and debilitating conditions in which the immune system loses tolerance to self-antigens and mounts an inappropriate defence against host tissues. They occur as a result of misdirected, self-directed immune response. They are defined as a clinical syndrome caused by the activation of immune cells in the absence of a foreign invader or another discernible cause [1]. Systemic Lupus Erythematosus (SLE) involves general inflammation caused by the accumulation of immune complexes in different organs and tissues. These complexes are formed when overactive B cells produce autoantibodies against the body’s own nuclear, cytoplasmic, and surface antigens [2]. Evidence shows that epigenetic changes in B cells, especially in promoter methylation at genes that control cell activation and survival, are directly informed in the loss of immune tolerance and ongoing autoantibody production in SLE [3]. Common symptoms include joint pain or arthritis, skin lesions, and involvement of multiple organ systems [4]. The frequency of SLE occurrence in the world is 40–50 persons per 100,000. Moreover, SLE is identified in women10-fold more often than in men, and this fact is most probably related to the presence of one hormone, estrogen. Rheumatoid Arthritis (RA), by contrast, is mainly a joint disease, marked by abnormal growth of the synovial tissue that leads to cartilage and bone damage, driven by persistent inflammation and the release of pro-inflammatory cytokines from activated B cells, T cells, and fibroblast-like synoviocytes (FLSs). The most prevalent clinical symptoms include swelling, pain, joint destruction, disability, and loss of function. FLSs play a crucial role in RA pathogenesis, which affects approximately 0.5% to 1.0% of the adult population [5].

Genetic predisposition significantly contributes to their etiopathology; however, an increasing body of research indicates that epigenetic dysregulation is pivotal in both the initiation and advancement of autoimmunity. Epigenetic modifications include reversible modifications that can be inherited which regulate gene expression without modifying the DNA sequence of the gene [6]. Global DNA hypomethylation in CD4+ T cells is characteristic of SLE. This anomaly results in the overexpression of self-reactive genes, such as lymphocyte function-associated antigen 1 (LFA-1), and an increased production of autoantibodies [7,8,9]. Moreover, genome-wide studies have highlighted concurrent alterations in histone modifications and non-coding RNAs in multiple immune cell subsets, further reinforcing the central role of epigenetic dysregulation in SLE [10]. In RA, imbalances in histone acetylation and methylation lead to the abnormal activation of FLSs, which enhances their characteristic invasiveness and accelerates cartilage and bone erosion [11,12,13]. These observations show that factors other than genetic predisposition influence the onset of autoimmune diseases. They are also heavily influenced by environmentally induced and fortunately reversible epigenetic modifications.

Despite remarkable advancements recorded by various research on biologic and targeted synthetic therapies, cure for both conditions remains elusive. Most current treatment strategies focus on managing symptoms, suppressing the immune system, and slowing the progression of the disease. While traditional pharmacological approaches, represented by 5-azacytidine, a DNA methyltransferase (DNMT) inhibitor, and trichostatin A, a histone deacetylase (HDAC) inhibitor, designed to rectify epigenetic anomalies, have demonstrated potential in preclinical autoimmune models, their clinical applicability is highly limited by poor disease specificity [14], significant toxicity [15], as well as off-target effects. Additionally, they have been shown to lack uniform effectivity as they work for some patients but not for others [16,17]. This, therefore, indicates the urgent need for new therapeutic approaches [11,12,18]. Similarly, traditional non-steroidal anti-inflammatory drugs (NSAIDs), disease-modifying anti-rheumatic drugs (DMARDs), and biologics, including corticosteroids, chemical DMARDs, biologic agents targeting B cells, T cells, and cytokines, as well as combinations such as methotrexate and TNF-α inhibitors, have yielded enhanced outcomes. But their high cost, incomplete responses, and side effects including infection, heart disease, osteoporosis, and liver toxicity, among others, limits their long-term use as a sustainable therapeutic approach to both SLE and RA [19].

Against this backdrop, researchers have continued to investigate plant-derived flavonoids, a class of polyphenolic compounds abundant in fruits, vegetables, and herbs, hoping to uncover effective, non-toxic, sustainable, and bioavailable therapeutic strategies for these ailments. Luteolin (LUT), quercetin (QUE), and apigenin (AP) are flavonoids that have been proven to have not only anti-inflammatory but also epigenetic-modulatory effects [20,21,22,23]. This makes them good candidates for new therapeutic interventions. In contrast to synthetic epigenetic medicines, these flavonoids are widely regarded as safe, cost-effective, and capable of simultaneously influencing many biochemical pathways [24,25,26,27,28].

Recent investigations support this promise, and this review aims to synthesize recent evidence regarding these flavonoids as epigenetic modulators in SLE and RA. LUT has demonstrated efficacy in mitigating lupus nephritis in mice models by regulating macrophage oxidative stress and re-establishing immunological equilibrium [29]. QUE has shown the capability to suppress IL-6 and TNF-α expression while activating the Silent Information Regulator 1 (SIRT1) pathway [30], which affects histone acetylation and improves immune modulation [31,32]. AP, on the other hand, has also demonstrated potency in stopping NF-κB signalling and changing how chromatin is arranged [23,33]. These results show that these flavonoids can function as natural epigenetic modulators, especially in SLE and RA, which is the scope of this review.

Flavonoids and polyphenols as a class of compounds, with a special focus on examples such as curcumin [31], resveratrol [34], and epigallocatechin gallate (EGCG) [35], which have all been the subject of dedicated mechanistic reviews centred on epigenetic pathways or autoimmune relevance. However, a focused comparative analysis of a potential therapeutic activity of luteolin, quercetin, and apigenin, specifically within SLE and RA, has not, to our knowledge, been sufficiently undertaken. Thus, the novelty of this present review lies in its disease-specific and mechanism-focused integration of epigenetic evidence for three flavonoids across two major autoimmune diseases. Rather than treating flavonoids as a general antioxidant class, this review maps LUT, QUE, and AP onto defined various epigenetic levels (DNA methylation, histone modifications, microRNAs, and chromatin-related signalling) and explicitly separates direct SLE/RA evidence from extrapolated mechanistic findings in other systems. In addition, it links these mechanisms to translational constraints such as dose, bioavailability, formulation, and the limited but emerging clinical evidence. Thus, it provides a more clinically interpretable framework than prior broad flavonoid or epigenetics reviews.

In this review, the epigenetic modifications characteristic for SLE and RA diseases are described, and the way flavonoids, namely LUT, QUE, and AP, influence DNA methylation and histone modifications, which are characteristic of these diseases, is described. Finally, translational progress, including limitations and future directions, will be evaluated. If larger clinical studies validate them, flavonoids may not only provide symptom relief but also address the epigenetic roots of autoimmunity. Preliminary evidence suggests that LUT, QUE, and AP may offer a valuable adjunct approach in the management of SLE and RA, which requires confirmation in further larger, disease-specific clinical studies.

### 1.1. Literature Search Strategy and Scope of the Review

The present work is a narrative, mechanism-focused review with a structured literature search of PubMed/MEDLINE, Scopus, Web of Science, and Google Scholar from database inception to March 2026. Search terms combined the compound names (“luteolin,” “quercetin,” “apigenin”) with epigenetic terms (“DNA methylation,” “histone acetylation,” “histone deacetylase,” “DNMT,” “microRNA,” “epigenetic”), and disease terms (“systemic lupus erythematosus,” “SLE,” “rheumatoid arthritis,” “RA,” “autoimmune”), and related synonyms using Boolean operators. We included original research articles, preclinical studies, clinical studies, and mechanistic reports relevant to the epigenetic actions of LUT, QUE, or AP in SLE and RA. Given the mechanistic and cross-disciplinary scope of the topic, mechanistic studies conducted in non-autoimmune cell systems (e.g., cancer cell lines) were also included, where they provided direct insight into epigenetic pathways relevant to SLE or RA. The final synthesis is narrative and emphasizes direct evidence from SLE and RA models, human autoimmune samples where available, and translational limitations. No formal risk-of-bias assessment or Preferred Reporting Items for Systematic Reviews and Meta-Analyses (PRISMA) framework was applied, consistent with the narrative structure of this review.

### 1.2. Distinct Immunopathological Features of SLE and RA

This review considers SLE and RA together under the broader theme of autoimmune epigenetic dysregulation; however, the two diseases differ substantially in their core pathology and dominant cell types. SLE is primarily driven by a breakdown of T- and B-cell self-tolerance, autoantibody production, immune complex deposition, and, in its most severe form, lupus nephritis [36]. RA, by contrast, is more strongly characterized by fibroblast-like synoviocyte (FLS) activation, synovial hyperplasia, macrophage-T-cell crosstalk, osteoclastogenesis, and pannus formation [37,38]. Where relevant, mechanisms discussed throughout this review are attributed to the specific cell type and disease model in which they were demonstrated (e.g., lupus-prone T cells versus RA-FLSs), rather than treated as generically interchangeable features of “autoimmune epigenetic dysregulation.” This distinction is important because a mechanism demonstrated in one cell type or disease context should not be assumed to apply directly to the other.

## 2. Mechanisms of Epigenetic Modifications by Flavonoids

The pathogenesis of both SLE and RA is associated with distinct, well-characterized epigenetic abnormalities that alter gene expression and promote autoimmunity. DNA hypomethylation is a key epigenetic anomaly common to these two diseases. This anomaly is driven largely by reduced activity of DNMT1 resulting from dysregulated microRNAs (notably miR-21 and miR-148a), oxidative stress, and aberrant histone modifications (particularly acetylation) that enforce an open chromatin state [8,39]. Additionally, the interplay of many epigenetic modification enzymes, especially those mediating histone modifications, plays a pivotal role in the onset and progression of autoimmune diseases [40,41]. Recent large reviews suggest that changes in DNA methylation and histone modification are not just side effects, but a common underlying problem in many autoimmune diseases, including SLE and RA [42]. In simple terms, these processes help decide how tightly DNA is packed and which genes are switched on or off. This control is handled by enzymes such as histone acetyltransferases (HATs), deacetylases, methyltransferases, and demethylases. When their activities are disrupted, immune cells can start behaving abnormally. The consequence is that the inflammatory genes may become too active, while genes that normally keep the immune system in check are weakened. This ultimately sets the stage for continuous immune activation and inflammation. For instance, when histone acetylation is poorly regulated, it can increase the activity of transcription factors like NF-κB, leading to higher cytokine production and promoting inflammatory immune cells, a pattern seen in RA models [40,41,43]. In a similar way, abnormal histone methylation, such as excessive activity of EZH2, can boost pro-inflammatory gene expression while diminishing regulatory mechanisms, helping to maintain long-term inflammation and autoimmune disease [40]. Therefore, therapies that would correct these anomalies would prove valuable in their treatment and cure.

Apart from histone deacetylation, other modifications of these proteins important for epigenetic regulations are known. They are performed by various enzymes such as histone methyltransferases (e.g., EZH2 and SUV39H1), histone demethylases (e.g., KDM family members), bromodomain proteins (e.g., BET/BRD4), and those introducing histone phosphorylation and ubiquitination [4,44,45].

**Figure 1:**
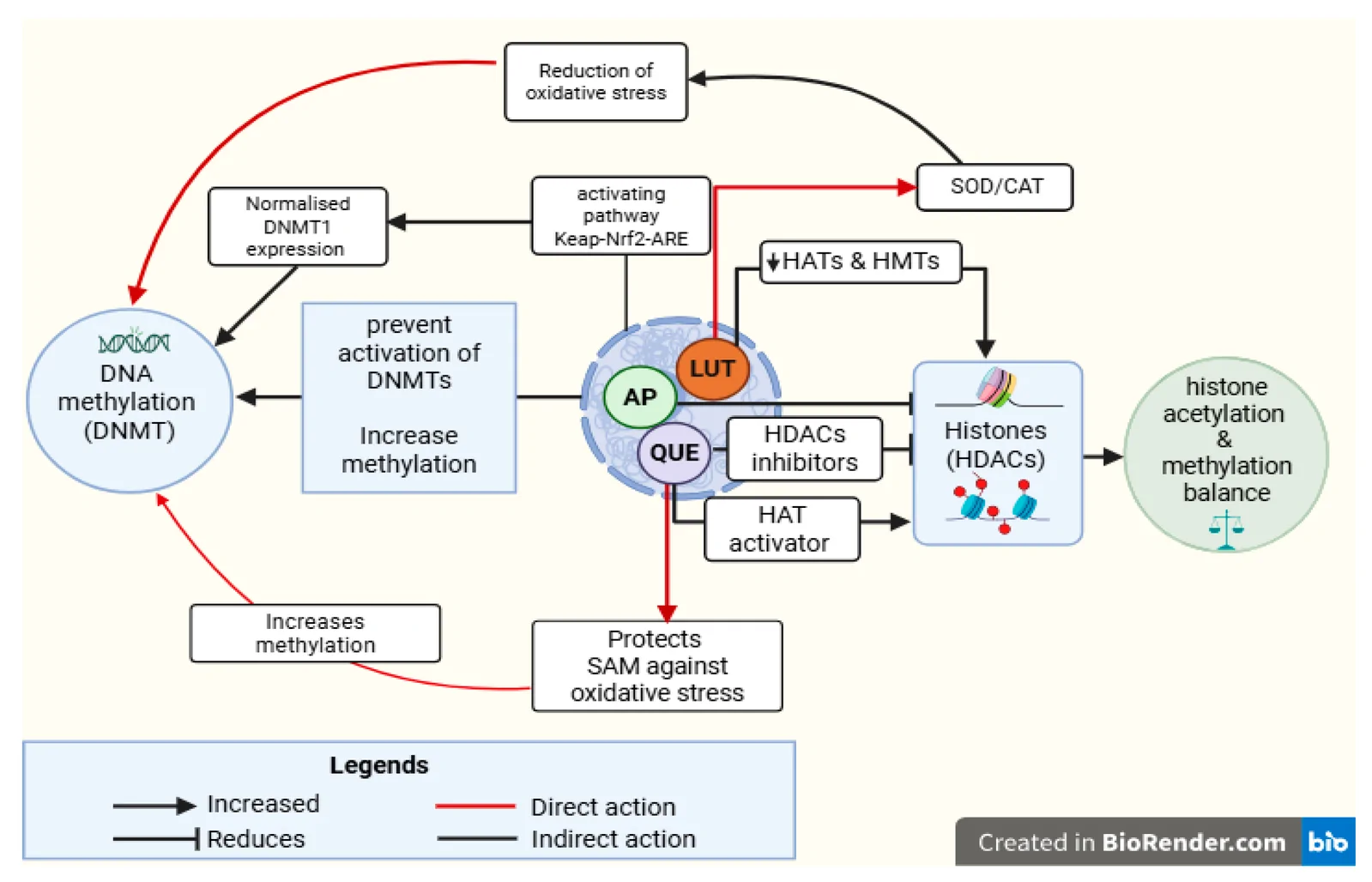
Epigenetic and immunomodulatory effects of AP, LUT and QUE in SLE and RA. Three principal functional domains targeted by these flavonoids in autoimmune diseases: DNA methylation through DNMT1, histone acetylation through HDAC inhibition, and antioxidant defence through induction of Superoxide Dismutase (SOD) and catalase (CAT) enzymes. The flavonoids converge on overlapping immune targets, including B cells, T cells, and cytokine networks, where they collectively reduce inflammatory signalling and restore immune balance. By simultaneously affecting epigenetic regulation and redox homeostasis, AP, LUT, and QUE may suppress pathogenic immune activation and support a shift toward a less inflammatory state in SLE and RA. The central overlap in the figure emphasizes their shared multi-target profile rather than a single linear pathway, highlighting the integrated nature of their epigenetic and immunomodulatory actions.

Flavonoids such as LUT, QUE, and AP have shown considerable promise as modulators of aberrant epigenetic alterations observed in autoimmune disorders (Figure 1). They have been shown to reverse abnormal gene expression patterns by directly interacting with and modulating the activity of essential epigenetic enzymes, such as DNMTs, HDACs, and Histone Methyltransferases (HMTs) [19,46,47]. They also act by indirect pathway modulation that alters DNMT stability, for example, the proteasome-mediated degradation or changes in transcription factors that control DNMT expression. These mechanisms produce promoter-specific epigenetic changes, thereby reactivating silenced genes in many cell models [24,47,48]. Table 1 summarizes epigenetic potency of AP, LUT and QUE in some relevant SLE and RA models.

Before examining LUT, QUE, and AP activity individually, it is important to make two important clarifications. First, descriptions of these flavonoids as both restoring DNMT1 activity/methylation and promoting demethylation could appear contradictory. These contradictions are only apparent, reflecting differences in the level of methylation being described rather than true inconsistency in compound action. Global DNA hypomethylation in autoreactive immune cells is a hallmark of SLE and, to a lesser extent, RA; in this setting, increased DNMT1 expression or activity may restore methylation at pathogenic loci in autoimmune cells, whereas reports describing flavonoids as DNMT or HDAC inhibitors that promote demethylation and reactivate silenced genes derive largely from non-autoimmune models, in which the baseline epigenetic state is different and the biological goal is gene reactivation [3,45]. Thus, the apparent contradiction reflects differences in disease context, target loci, and cell type rather than a single uniform direction of action. Throughout this review, effects are therefore described as global, gene/promoter-specific, or cell-type-specific wherever the underlying data allow this distinction. Second, and related to the foregoing, a substantial proportion of the mechanistic evidence discussed below, particularly for HDAC inhibition and chromatin remodelling, was generated in non-autoimmune cell systems such as cancer or leukemia cells, rather than directly in RA-FLSs, SLE T cells, B cells, or monocytes. These findings provide important biochemical proof of concept for how each compound can engage DNMTs, HDACs, and related enzymes, but they should not be interpreted as direct demonstrations of efficacy in SLE or RA. Evidence obtained directly from SLE- or RA-relevant models, including lupus-prone mice, collagen-induced arthritis models, RA-FLSs, and patient-derived samples, is distinguished from such extrapolated mechanistic evidence throughout the text and in Table 1.

**Figure 2:**
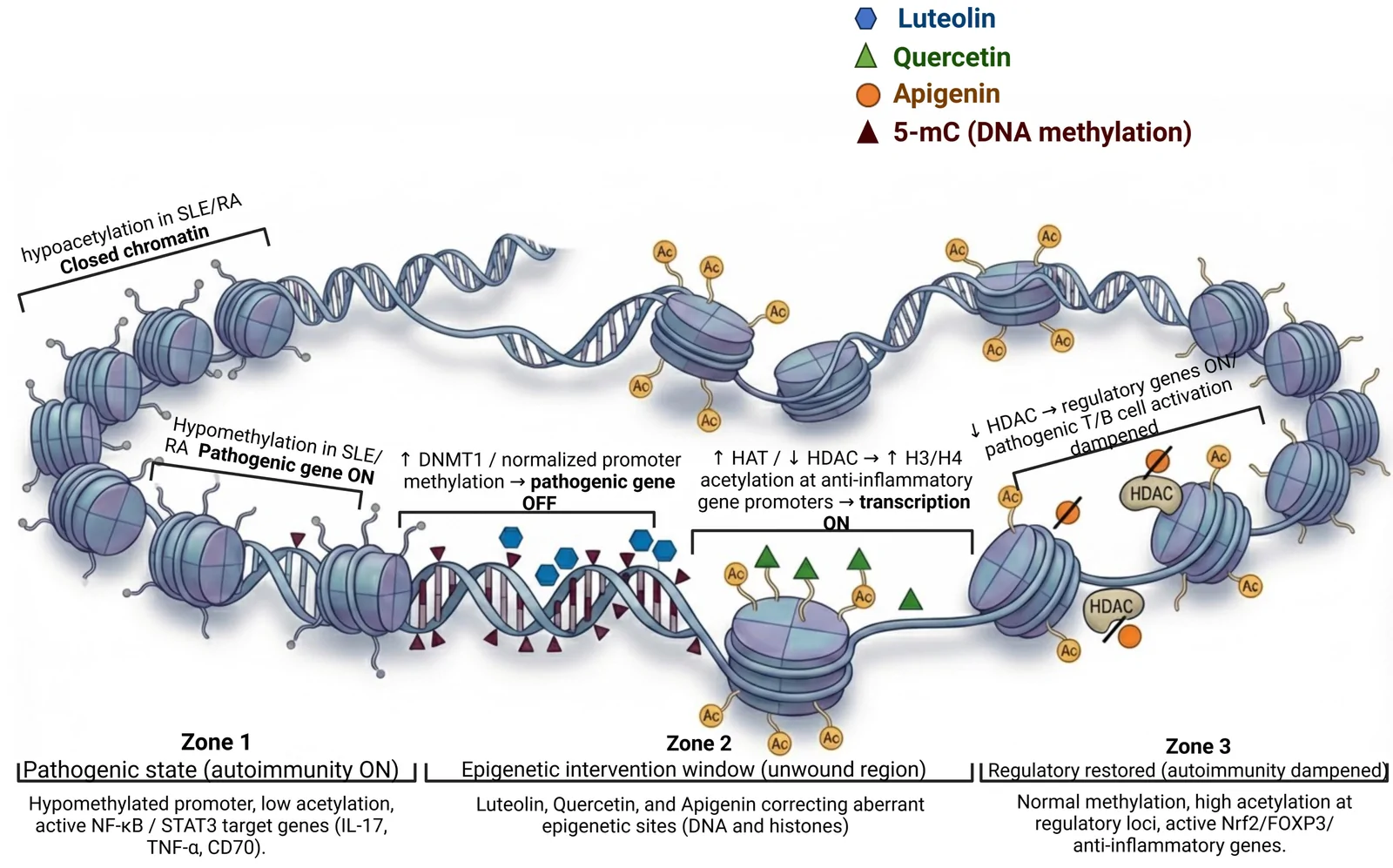
Sites of epigenetic modification effects of AP, LUT, and QUE (created in www.BioRender.com, repronted/adapted with permission from Ref. [76]). The figure illustrates a three-zone model, in which SLE and RA begin in a hypomethylated, low-acetylation, pro-inflammatory chromatin state characterized by some active additional genes and increased NF-κB/STAT3 signalling. In the central intervention window, LUT, QUE, and AP counter these abnormalities by restoring DNMT1-dependent promoter methylation, increasing HAT-mediated histone acetylation, and inhibiting HDAC activity at anti-inflammatory loci. This shifts chromatin toward a more open, transcriptionally permissive state for regulatory genes while dampening pathogenic T- and B-cell activation. In the final zone, the combined epigenetic effects are represented as a restored regulatory state with a normalized methylation level, increased Nrf2/FOXP3/anti-inflammatory gene activity, and decreased autoimmune signalling in SLE and RA.

These flavonoids are known to increase DNA methylation by DNMTs or by preventing oxidative inactivation of these enzymes. These two pathways tend to affect each other since cellular redox imbalance affects methyl group donors, S-adenosylmethionine (SAM), resulting in DNA hypomethylation [67]. LUT, QUE, and AP converge on the nuclear factor erythroid 2-related factor 2 (Nrf2) signalling pathway, which affects redox homeostasis and antioxidant response element (ARE)-driven gene expression [68]. They possess antioxidant properties to stabilize redox imbalance and by extension stabilize DNA methylation processes. Studies show that LUT indirectly normalizes DNMT1 expression by activating the Keap1–Nrf2–ARE pathway. This route restores cellular redox equilibrium, induces endogenous antioxidant defences (e.g., SOD, Heme Oxygenase 1 (HO-1), NAD(P)H: Quinone Oxidoreductase 1 (NQO1)), and mitigates reactive oxygen species (ROS). This achieves the alleviation of oxidative stress, which suppresses and destabilizes DNMT1 [68,69]. Various studies have demonstrated the potency of QUE in facilitating normal DNA methylation by protecting SAM against oxidative depletion and suppression [60,67]. QUE preserves the functions of methionine synthase (MS) and cystathionine β-synthase (CBS), key enzymes in homocysteine and SAM metabolism, by decreasing ROS through Nrf2 pathways [70,71,72]. Just like LUT and QUE, AP modulates aberrant histone modification through numerous mechanisms, including free radical scavenging, metal chelation, and regulation of redox signalling pathways (NF-κB, Nrf2, MAPK, PI3K/Akt) [63,73,74,75] (Figure 2).

In immune cells, HDACs, especially upregulated HDAC2, HDAC7, and altered class I/II HDACs, promote the expression of pro-inflammation genes and other mediators of autoimmunity [77]. Research has shown however that these flavonoids can correct abnormal histone modifications associated with severe autoimmune disorders. LUT and AP are known HDAC inhibitors. By suppressing the activity of notable HDACs (HDAC1, HDAC2, HDAC3, HDAC5, HDAC7, HDAC10, HDAC11), they alter histone acetylation and methylation patterns [23,50,78,79]. LUT downregulates HATs and HMTs, leading to normalized histone acetylation and methylation balance, discouraging the abnormal chromatin states (e.g., excessive H3K27me3 or H3K9 hypomethylation) found in autoimmune states [50]. QUE, although a weak HDAC inhibitor, acts as a good HAT activator for histone acetylation, especially H3. QUE’s promotion of histone H3 acetylation is closely linked with activation of mitogen-activated protein kinase (MAPK) signalling pathways, notably the extracellular signal-regulated kinase (ERK) and c-Jun N-terminal kinase (JNK) cascades, which regulate diverse transcription factors such as c-Jun and activator protein-1 (AP-1) [68]. These are key signalling pathways inside the cell. For instance, in HATs such as p300/CBP, in order for them to function properly, QUE triggers these phosphorylation events by activating the ERK and JNK pathways. This in turn upregulates histone H3 acetylation (H3K9ac) [68,80]. The effect is the promotion of an epigenetically permissive chromatin state that enhances gene transcription [80,81].

## 3. Mechanisms of Epigenetic Modulation by LUT, QUE and AP

### 3.1. Mechanism of Epigenetic Modulation by LUT in SLE and RA

**Figure 3:**
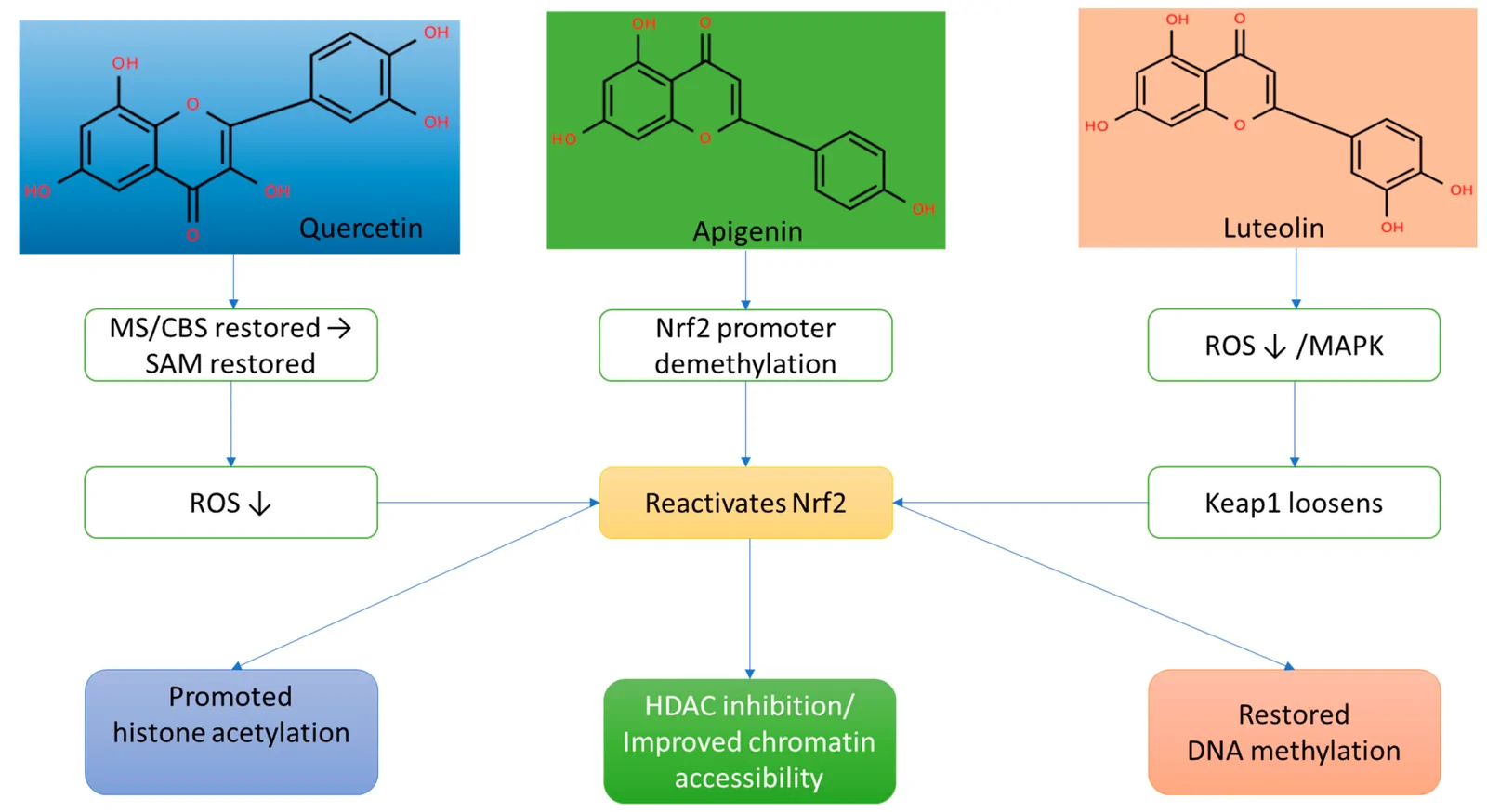
Summary of key pathways for epigenetic modifications by AP, LUT, and QUE. This scheme shows how the three flavonoids converge on Nrf2 reactivation through partially distinct upstream mechanisms. QUE reduces ROS and restores MS/CBS activity, thereby replenishing SAM and supporting DNA methylation, while also favouring downstream histone acetylation. AP promotes Nrf2 promoter demethylation, which facilitates Nrf2 reactivation and subsequent chromatin relaxation through HDAC inhibition. LUT decreases ROS and attenuates MAPK signalling, loosening Keap1-mediated repression of Nrf2. Collectively, these pathways restore DNA methylation balance, improve chromatin accessibility, and support antioxidant and anti-inflammatory gene expression.

The flavonoid LUT (3′,4′,5,7-tetrahydroxyflavone; C_15_H_10_O_6_) has been shown to possess anti-inflammatory, antioxidant and DNA methylation modulation effects [82]. This makes it at once a palliative agent and a potential therapeutic strategy to SLE and RA diseases (Figure 3). Among other mechanisms, LUT upregulates DNMT1 expression by decreasing oxidative stress [82,83,84] and levels of miRNAs (such as miR-21 and miR-148a) that directly or indirectly inhibit DNMT1 [39]. From cell-based experiments on animal disease models and early human data, LUT consistently targets the epigenetic machinery dysregulated in SLE and RA. Its multi-pronged action, including restoring balance in methylation, inhibiting HDACs, and balancing HAT/HMT activity, has been associated with preclinical normalization of disease-associated chromatin states and reduced inflammatory pathology in the cell and animal models discussed below, supporting further investigation of LUT as an adjunct compound in epigenetic-directed strategies for autoimmunity. Recent research has shown that LUT treatment corrects hypomethylation at key gene loci including CD70 and CD11A in T cells by elevating DNMT1 expression. This way, it reverses the overexpression associated with autoreactivity in SLE. Experimental models have demonstrated that LUT downregulates the MAPK/ERK pathway, which normally suppresses DNMT1, hence restoring a proper methylome [45,49]. In studies with the use of nuclear extracts from HeLa cells, a cervical cancer cell line unrelated to SLE or RA, LUT exhibited a dose-dependent suppression of overall DNMT activity, decreasing it by 35%, 57%, and 66% at concentrations of 5, 10, and 20 µM, respectively, thereby reactivating genes suppressed by methylation in this cancer model. LUT also facilitated demethylation at 5′-CpG islands within gene promoters in this system, reinstating the transcription of immune-regulatory and anti-apoptotic genes. While this HeLa-derived evidence is not itself autoimmune disease data, the affected gene classes are significantly pertinent in SLE and RA, where hypomethylation induces autoimmune activation [50]. The fact that LUT inhibits DNMT activities and promotes demethylation might appear counterintuitive for SLE and RA marked by improper state of DNA hypomethylation. This apparent paradox, however, reflects the complexity and site-specific nature of DNA methylation in autoimmune diseases and the dual, regulatory impact of both demethylation and remethylation on gene expression.

The inhibitory activities of LUT on HDAC have been demonstrated using both immune and non-immune cell lines. In in vitro research using human and murine cell lines, LUT directly inhibits the enzymatic activity of HDACs in a dose-dependent manner, with a decrease of 28%, 40%, and 55% at concentrations of 5, 10, and 20 µM, respectively [50]. The inhibition is associated with the down-regulation of gene expression for various HDACs, including HDAC1, HDAC2, HDAC7, HDAC10, and HDAC11, as well as a decrease in HAT and HMT (H3K9) activity [50]. The modifications improve the acetylation of histones H3 and H4 (e.g., H3K9ac, H3K18ac), resulting in chromatin relaxation and induction of expression of genes related to immune regulation [24,50]. LUT treatment in cell models representative of SLE/RA has resulted in a significant reduction in pro-inflammatory cytokines (IL-6, TNF-α) and an elevation of proteins encoded by protective genes including p53 and E-cadherin [50].

LUT induces significant epigenetic effects in animal models, with reported anti-inflammatory and disease-modifying outcomes in these preclinical systems. In animal models of RA, LUT inhibited inflammatory cytokines by modulating histone acetylation, specifically through inhibition of HDAC activity. Furthermore, this flavonoid improved immune tolerance by altering the T-cell repertoire to reduce pathogenic Th17 phenotypes [44,85]. LUT attenuated RA-associated chronic pain by targeting the LDHA/H3K9la/NFATC2 axis to suppress Th17 cell differentiation. In collagen-induced arthritis (CIA) in mice, daily intraperitoneal injections of a formulation containing LUT, frequently combined with palmitoylethanolamide (PEA+LUT) at a dosage of 1 mg/kg for 10 days, led to significant reductions in joint erythema, swelling, and histological damage when compared to control (untreated CIA mice) [51]. The intervention enhanced cartilage integrity and reduced the infiltration of inflammatory cells in the joints. Also, plasma concentrations of pro-inflammatory cytokines (TNF-α, IL-1β) and indicators of oxidative and nitrosative damage (malondialdehyde, nitrotyrosine) were significantly diminished [51]. The treatment with LUT decreased HDAC activity and restored HAT/HDAC balance, as demonstrated by the chromatin immunoprecipitation (ChIP) method in splenic and peripheral immune cells. This resulted in an increase in histone acetylation at anti-inflammatory gene promoters, thereby reinstating their expression [51,52]. In a study by Jiang and coworkers [85], LUT not only reduced joint pain but also changed immune and glial activity in the spinal cord of CIA mice. LUT limited spinal CD4^+^ T-cell infiltration and microglial activation. Multi-omics analyses performed identified Nuclear factor of activated T cells 2 (NFATC2) as a key transcription factor driving Th17 differentiation and spinal entry via a Protein Kinase C Epsilon (PRKCE)–Signal Transducer and Activator of Transcription 3 (STAT3) signalling axis. Mechanistically, LUT directly bound and inhibited Lactate dehydrogenase A (LDHA), decreased glycolysis-derived lactate and lactylation of the histone H3 in a lysine 9 position (H3K9la), which epigenetically silenced NFATC2 and curtailed Th17 pathogenicity. The same LDHA/H3K9la/NFATC2 pathway was hyperactive in CD4^+^ T cells of RA patients and could be normalized ex vivo by LUT [85].

Furthermore, studies with lupus-prone mice, particularly the Murphy Roths Large/lymphoproliferation strain (MRL/lpr) and New Zealand Black/New Zealand White first filial generation hybrid (NZB/NZW F1) strains, indicate that a daily injection of LUT (25–100 mg/kg) led to substantial reductions in joint inflammation, autoantibody titres, and immune cell infiltration in renal and synovial tissues [29]. MRL/lpr mice exhibit hypomethylation at the promoters of genes associated with pathogenic immune activation, such as CD11A, CD70, and IL-17A. LUT treatment normalized DNA methylation profiles by enhancing DNMT1 activity through the restoration of ERK/MAPK signalling, thereby reversing pathogenic hypomethylation [9,29,53]. LUT, similarly to other HDAC inhibitors, reinstated the expression of essential regulatory miRNAs, including the Dlk1-Dio3 cluster, which are epigenetically silenced in lupus nephritis (LN) [29,86]. ChIP analysis combined with omics analyses revealed that LUT influenced the equilibrium between HATs and HDACs, facilitating chromatin structures that favour transcriptionally active states, thereby enhancing anti-inflammatory and pro-apoptotic gene expression [52]. The molecular and epigenetic changes were significantly associated with improved physiological outcomes and a notable decrease in lupus severity.

### 3.2. Mechanism of Epigenetic Modulation by QUE in SLE and RA

QUE (C_15_H_10_O_7_), also known as 2-(3,4-dihydroxyphenyl)-3,5,7-trihydroxychromen-4-one, is a prevalent flavonoid found in various dietary sources, including vegetables, fruits, and wine. This substance is recognized for its antibacterial and antioxidant properties [58]. In the last ten years, it has attracted interest in epigenetic research due to its capacity to influence epigenetic mechanisms in autoimmune diseases, especially SLE and RA (Figure 3). The effect of QUE on DNA methylation and DNMT modulation in human immune cell lines, specifically peripheral blood mononuclear cells (PBMCs), T cells, and monocytes, was examined. These cells were cultured and treated with QUE at varying concentrations from 5 to 100 μM over a duration of 24 to 72 h. The results showed that treatment with QUE significantly decreased DNMT1 and DNMT3A/B at both the mRNA and protein levels [54,55,87]. In cellular models of SLE and RA, QUE efficiently rectified aberrant methylation patterns at the promoters of crucial immune-regulatory genes, such as encoding IL-2, FOXP3, and p16. This restoration of gene expression facilitated the re-establishment of immunological tolerance and the reduction in pro-inflammatory gene expression [54,57,87]. Also, Lee et al. (2011) [56] conducted mechanistic experiments related to human leukemia, a non-autoimmune malignancy. They used the HL-60 cell line to show how QUE impacts the addition of acetyl and methyl groups to histones. QUE was added to HL-60 cells at concentrations between 25 and 100 μM for durations of 6 to 12 h. A dose range of 75–100 μM significantly enhanced H3 acetylation at the FasL promoter after 3–12 h, leading to an increased expression of this gene and apoptosis in this leukemia model [56]. FasL is a Fas ligand, which primarily triggers apoptosis, regulates the immune system, and maintains immune privilege in specific tissues by binding to the Fas receptor. While direct confirmation of this mechanism in SLE or RA immune cells is currently lacking, this example shows that QUE can affect the expression of some genes related to modifications of histones and chromatin. The increased FasL expression and apoptosis could suggests a route by which QUE could help rebalance aberrant immune cell behaviour.

Recently, the effect of QUE on miRNA expression in senescent CD4+ T cells from lupus-prone MRL/lpr mice, FLSs, and human PBMCs was examined. In FLSs produced from RA, QUE increased the level of an anti-inflammatory microRNA (miR-146a), while simultaneously inhibiting GATA6, resulting in reduced cell migration and invasion. The results exhibited a down-regulation of pro-inflammatory cytokines (IL-1β, TNF-α, IL-6) and an inhibition of the NF-κB and MAPK pathway, all of which are closely linked to the epigenetic regulation of inflammatory genes [58]. In senescent CD4+ T cells from lupus-prone MRL/lpr mice and human PBMCs, an up-regulation of miR-146a and other anti-inflammatory miRNAs, alongside a down-regulation of miR-21 and miR-27a, was observed [59,88]. The expression of miRNAs is regulated by DNA methylation and histone modifications, which are known to be modulated by QUE [54,56,58,59]. QUE influences miRNA profiles through its extensive epigenetic effects on DNA and histone-modifying enzymes, serving as a mechanism to refine immune responses in autoimmunity. Molecular docking and dynamics simulations predicted that QUE binds to DNMTs, HDACs, and SIRT1/6. Kedhari and coworkers employed Auto Dock along with various computational techniques to dock QUE into the active sites of DNMT1 and HDAC1/2 [57]. The findings indicate that QUE has a direct inhibitory effect by establishing hydrophobic interactions and stable hydrogen bonds with critical amino acid residues located in the catalytic regions of these enzymes [57]. These studies, combining network pharmacology and molecular docking techniques, show a potential role of QUE in autoimmune diseases. Here, QUE was tested against key inflammatory signalling proteins and epigenetic enzymes linked to RA and SLE. This compound was potentially able to interact with the amino acid residues in active sites of the enzymes needed for their function, showing strong binding affinity [87]. However, these results are computational prediction, which need further experimental confirmation in disease-relevant cells and in vivo models.

Results of studies using animal models suggest that QUE’s epigenetic effects are partly driven by changes in microRNAs, including increased miR-146a and reduced miR-21 and miR-27a (Table 1 and Table 2), alongside effects on major signalling pathways such as NF-κB, Nrf2, MAPK, and HO-1, all of which influence regulation of gene expression and chromatin structure [58,62,89]. In lupus-prone mice and arthritis models treated with QUE for several weeks, researchers observed lower proteinuria, reduced autoantibody levels, and less immune cell infiltration in affected organs, together with restored DNMT1 activity, proper DNA methylation level at disease-related genes like CD70 and IL-17A, and improved histone acetylation at anti-inflammatory sites [58,59].

Although limited, early clinical and translational studies are beginning to support these findings. In RA, patients receiving QUE-rich diets or supplements for 8 to 12 weeks showed reduced expression of HDACs and DNMT1, improved DNA methylation patterns, more proper miRNA profiles, and lower inflammation, without notable side effects [60,62]. In SLE, while direct trials remain scarce, dietary and translational studies suggest that QUE may reduce DNMT expression and restore the proper methylation level of some regulatory genes such as IL2 and FOXP3, helping to improve immune balance and reduce autoantibody production [9,93,94]. Another interesting data from a recent clinical trial studies indicates that QUE supplementation can slightly shift the expression of genes linked to inflammation, oxidative stress, and metabolic regulation [61,88]. Similarly, Ou and colleagues, in a randomized controlled trial, observed a fairly modest reduction in inflammatory biomarkers, precisely C-reactive protein (CRP) and IL-6, especially in individuals with chronic diseases or in those treated with higher doses of QUE [61]. Altogether, the findings show that QUE may exert therapeutic effects through epigenetic pathways. The above results are reinforced by the discovery that QUE increases the levels of anti-inflammatory miRNAs (like miR-146a) and decreases the levels of pro-inflammatory miRNAs (like miR-21 and miR-27a) in RA patient models. This leads to decreased cytokine production and lower immune cell activation [62,89].

In clinical terms, these epigenetic alterations attributed to QUE result in substantial decreases in disease activity ratings (DAS28 and HAQ), pain levels, and inflammatory markers (CRP, TNF-α, IL-6) in RA, without notable side effects. In SLE, observational and dietary intervention studies indicate diminished autoantibody titres, enhanced renal function, and improved immune cell profiles, all associated with the epigenetic reprogramming of immune cells [58,59]. Despite these potentials, more standardized and mechanistically focused trials are needed to confirm how reliably QUE can reshape the epigenetic landscape in humans.

### 3.3. Mechanism of Epigenetic Modulation by AP in SLE and RA

Attention of researchers has also turned to the epigenetic modulation potential of AP, 4′,5,7-trihydroxyflavone, which is commonly found in vegetables, fruits or plants, particularly parsley, celery seeds, and chamomile flower [95,96,97]. It has relatively low toxicity on normal cells and cells with biochemical alterations [95]. While most of studies were performed on cancer cells, in vivo studies showed that AP can directly or indirectly impact epigenetic pathways that are dysfunctional in autoimmune diseases (Figure 3). In RA-FLSs, Shin and colleagues [98] demonstrated that AP triggers robust, ROS-dependent activation of ERK1/2, which in turn activates caspase-3/7 and drives apoptosis of these hyperproliferative synovial cells. Although this work did not directly measure histone marks, it establishes RA-FLSs as a disease-relevant target of AP-induced cell death [98]. Complementary mechanistic studies in human prostate cancer cells by Pandey et al. [23] showed that AP at concentrations of 20–40 μM selectively inhibits class I histone deacetylases, particularly HDAC1, with associated increases in global and promoter-specific histone H3/H4 acetylation at loci such as p21, leading to chromatin relaxation, cell-cycle arrest, and apoptosis, reduction in the Bcl-2/Bax ratio, and more importantly to this review, a significant suppression of key inflammatory mediators such as TNF-α and IL-6 [32,96]. It is important to note that the pro-apoptotic, ROS/ERK1/2-dependent effect of AP was demonstrated directly in RA-FLSs, whereas the epigenetic mechanisms such as HDAC1 inhibition and H3/H4 acetylation at p21 that affect chromatin remodelling were defined in a non-autoimmune, non-synovial model (i.e., human prostate cancer cells). This mechanism has not yet been directly confirmed in RA-FLSs, SLE T cells, B cells, or monocytes, and its extrapolation to RA synovial biology has to be validated. These findings suggest AP, which combines directly demonstrated pro-apoptotic signalling in RA-FLSs with the HDAC-inhibitory and chromatin-remodelling actions defined in other cell types, as a potential epigenetic modulator candidate capable of reshaping synovial pathogenic programmes in RA. If the HDAC-inhibitory mechanism observed in cancer cells is confirmed directly in RA-FLSs, restoration of gene function, enhancement of apoptosis of hyperproliferative synoviocytes, reduction in synovial inflammation, and normalization of immune responses could plausibly follow from the ability of AP to increase histone acetylation and reverse aberrant silencing of the expression of anti-inflammatory and cell cycle arrest genes, which would be directly relevant to the core pathogenic features of RA, characterized by hyperproliferation, overexpression of HDAC1, HDAC2, and HDAC11, and persistent inflammatory cytokine signalling [32,98,99,100].

**Figure 4:**
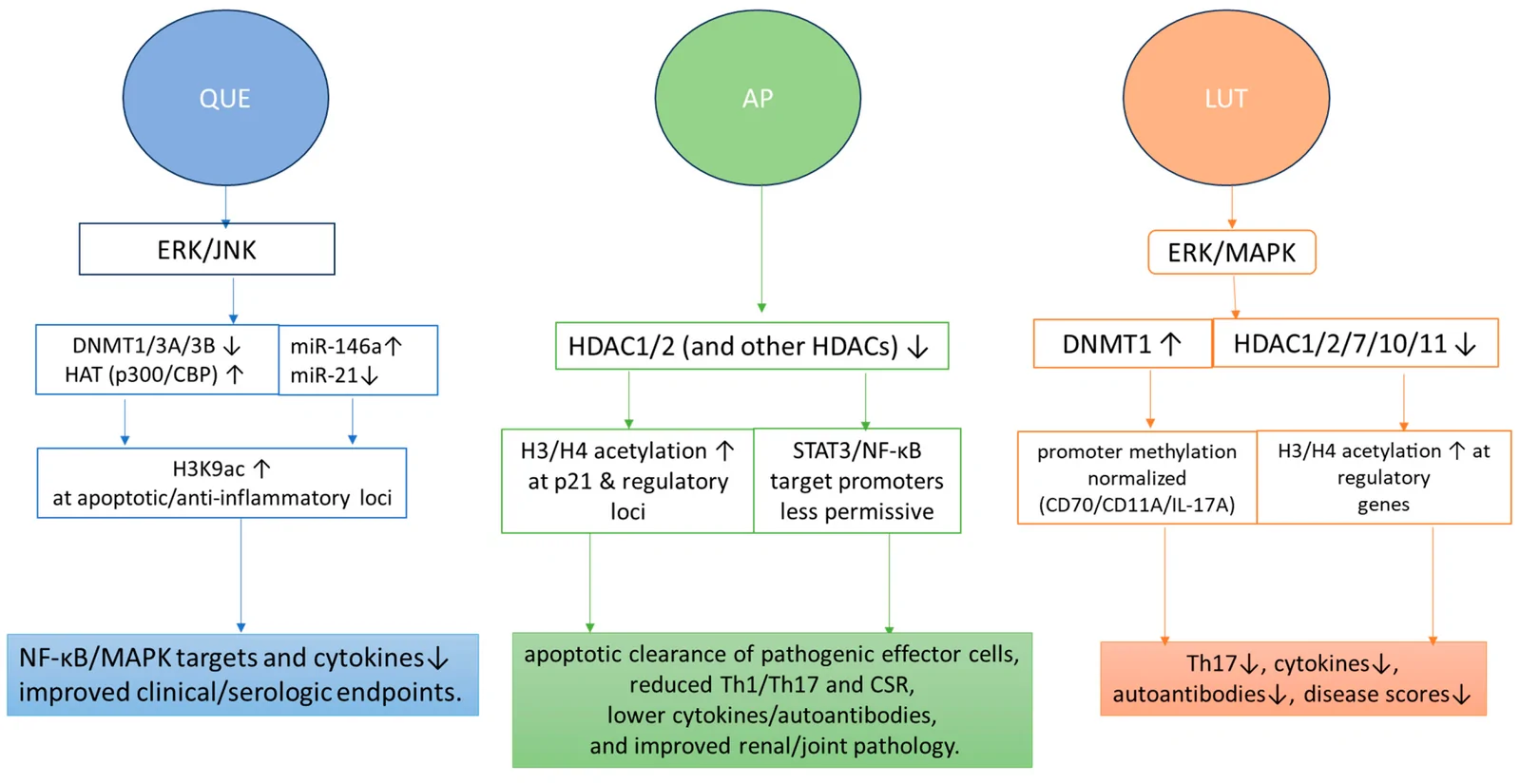
Post-Nrf2 epigenetic modification pathways of AP, LUT, and QUE. The figure outlines the main downstream epigenetic effects of AP, LUT, and QUE after activation of Nrf2-linked signalling. QUE acts mainly through ERK/JNK-associated pathways, modulating DNMT1/3A/3B, HATs such as p300/CBP, and miR-146a/miR-21, which promotes H3K9 acetylation and suppresses NF-κB/MAPK-driven inflammatory signalling. LUT acts largely through ERK/MAPK-mediated restoration of DNMT1 and inhibition of HDAC1/2/7/10/11, leading to normalized promoter methylation at disease-associated loci and reduced Th17 responses, autoantibody production, and tissue injury. AP inhibits HDAC1/2 and related HDACs, increases H3/H4 acetylation at regulatory promoters, and can also promote CpG demethylation at the Nrf2 promoter, supporting antioxidant and immunoregulatory gene expression. Altogether, these pathways converge on reduced inflammation, enhanced apoptosis of pathogenic cells, and improved renal and joint outcomes in SLE and RA.

AP also showed potential anti-arthritic and epigenetic effects in adjuvant Complete Freund’s Adjuvant (CFA) and CIA rat and mouse models [32,64,65]. The CFA rats after treatment with AP exhibited improved arthritis scores, as well as significantly decreased levels of pro-inflammatory cytokines, namely IL-1β, IL-6, and TNF-α in serum and tissues [32,64]. In another study using CIA mice, dendritic cells treated with AP showed a reduced ability to stimulate pathogenic T-cell responses, which resulted in a lower cytokine production and inflammatory cell infiltration in joints [32,65]. All these cells demonstrated a strong immunosuppressive and anti-inflammatory activity of AP through an epigenetic modulation involving suppressed expression of HDACs and remarkable increase in global and promoter-specific acetylation of H3 and H4 in immune target tissues. The overall epigenetic modification impacts include histone hyperacetylation favouring open chromatin states at anti-inflammatory and immunoregulatory gene loci, restored gene accessibility and transcriptional activation required for immune regulation, thereby decreasing pathogenic T-cell activation and cytokine levels [32]. Additionally, a mechanistic study using mouse skin epidermal JB6 P+ cells (i.e., a non-immune, non-autoimmune model), showed that AP restored the silenced Nrf2 antioxidant pathway by promoting CpG demethylation at the Nrf2 promoter in a dose-dependent manner that increased Nrf2 expression [63] (Figure 4). This enhanced tissue resilience against oxidative stress as well as helped to regulate inflammation through redox and epigenetic control [101], showing a link between immune regulation and cellular stress adaptation.

The epigenetic potential of AP in some relevant SLE and RA models is summarized in Table 1. Generally, AP was shown to inhibit HDAC activity while increasing histone acetylation at regulatory loci, including genes crucial for immune tolerance. Further investigations using immune cells transfected with Nrf2-luciferase reporter constructs showed that AP not only enhanced Nrf2 transcriptional activity but also induced CpG demethylation at the Nrf2 promoter [32]. This epigenetic regulation of the Nrf2 expression led to improved cellular defence against oxidative stress, reduced apoptosis, and better cell survival [19,32]. Thus, AP both epigenetically and transcriptionally affected the antioxidant and cytoprotective Nrf2 pathway. These modulations are particularly important in SLE, in which oxidative stress and immune cell death fuel systemic autoimmunity.

Classical LN readouts like antinuclear antibodies (ANA), anti-dsDNA, cytokines, and renal histology were significantly improved by AP in the MRL/lpr lupus-prone-mice study [33,66]. Additionally, by inhibiting STAT3/IL-17 signalling and promoting CD8^+^ T-cell apoptosis, AP reduced renal infiltration of CD8^+^ cytotoxic T lymphocytes [66,102,103]. Single-cell RNA-seq data from human LN kidneys identified STAT3-regulated CTLs as the dominant CD8^+^ subset, supporting the translational relevance of this mechanism [66,104,105]. These findings strengthen the hypothesis that AP can epigenetically and transcriptionally reprogram pathogenic CD8^+^ T-cell responses in LN and support its potential as a dietary adjunct in SLE.

Flavonoids, such as LUT, QUE, and AP, are moving from purely experimental epigenetic tools toward nutraceuticals with genuine translational potential in autoimmune disease, but their clinical use remains limited by formulation, dosing, and trial design issues [55].

### 3.4. MicroRNA and Long Non-Coding RNA Regulation by LUT, QUE, and AP in SLE and RA

Non-coding RNAs have emerged as important post-transcriptional regulators in autoimmune diseases, with dysregulated microRNAs (miRNAs), long non-coding RNAs (lncRNAs), and circular RNAs (circRNAs) that contribute to synoviocyte activation, immune cell signalling, cytokine production, and apoptosis. Based on the literature data presented here, the most substantial evidence currently concerns miRNA-mediated effects. In particular, QUE modulated the miR-146a/GATA6 axis in RA-FLSs, thereby suppressing migration and invasion, and regulating the miR-431-5p/MDM4 pathway, with associated inhibition of invasion and promotion of apoptosis [90,91]. This compound also inhibited production pf inflammatory cytokines, while it suppressed the lncRNA XIST and engaged a miR-485/PSMB8-associated mechanism [92]. LUT was linked in SLE-related models to restoration of the transcriptional activity of the epigenetically silenced Dlk1-Dio3 miRNA cluster, supporting conformation of a broader role for flavonoids in ncRNA regulation. Table 2 shows data for the ncRNA-related findings and distinguishes direct evidence obtained in flavonoid-treated SLE or RA models from mechanistic or inferred associations.

Although lncRNAs and circRNAs are increasingly recognized as contributors to SLE and RA pathogenesis, direct evidence linking LUT, QUE, or API to lncRNA or circRNA regulation specifically in these diseases remains limited. This is an important gap in the literature, which can be explored in future studies.

## 4. Clinical Prospects

Preclinical work clearly shows that LUT, QUE, and AP can normalize DNMT1 activity, correct disease-linked promoter methylation, inhibit HDACs, increase H3/H4 acetylation at regulatory loci, and rebalance miRNAs such as miR-146a and miR-21, with parallel improvements in cytokines, autoantibodies, nephritis scores, and arthritis severity [39,58,62,89]. Early human data suggest that QUE can downregulate HDAC1/2/11 and DNMT1 in PBMCs and improve global methylation and miRNA profiles, but equivalent human data for LUT and AP are still lacking [55,60,99]. Nutraceutical and anti-inflammatory trials with flavonoids already exist in related inflammatory conditions. However, among the three flavonoids, only QUE has been used in clinical trial in our select autoimmune diseases and these trials are scarce and basically RA-specific [26,61,62,106]. A randomized double-blind trial of QUE, 500 mg/day for 8 weeks, was conducted among/recruited 50 women with active RA. The results obtained show significant reduction in early morning stiffness, pain (both in the morning and after activity), and tender joint count compared with placebo, and improved disease activity score (DAS28) with *p* values around 0.001 for key outcomes. Inflammatory markers were also improved, with a significant drop in high-sensitivity TNF-α and better erythrocyte sedimentation rate (ESR), and no important adverse effects were observed, indicating good short-term tolerability [26,60]. Similar results were obtained by Prananda et al. (2024), who conducted randomized and observational studies using QUE doses from 45 to 2000 mg/day in rheumatological conditions, including RA [62]. In most studies, supplementation with QUE resulted in significant reduction in pain, morning stiffness, DAS28 scores, and inflammatory biomarkers such as CRP and IL-6, along with improved quality of life [62]. Observational and dietary interventions in SLE and other chronic diseases reported a modest decrease in CRP and IL-6 levels [61] and improved renal or immune parameters when QUE-rich regimens were used [59]. Literature data support the feasibility of moving from generic nutraceutical use to targeted pilot autoimmune trials. However, currently, studies in this issue are scarce, heterogeneous in respect to used dose and type of formulation, and rarely incorporate direct epigenetic endpoints. Thus, while they have proven the principles, they are not yet to be seen as definitive therapy.

Several limitations constrain the interpretation of these clinical data, which have to be stated explicitly. In respect to LUT, QUE, and AP, completed clinical trials in SLE or RA remain scarce. To date, only QUE has been evaluated in randomized trials specifically in RA patients, while completed trials for LUT and AP in either SLE or RA and their translational evidence rest entirely on preclinical models. Even the available QUE trials are constrained by small sample sizes (typically fewer than 60 participants), short follow-up periods (8–12 weeks), and non-standardized doses and formulations (ranging from approximately 45 to 2000 mg/day across studies), which limits comparison of the results between individual trials. None of the completed clinical studies incorporated direct epigenetic endpoints (e.g., DNMT or HDAC activity, locus-specific methylation, or miRNA profiling) alongside clinical outcomes. Consequently, while preclinical work links QUE’s clinical benefits to epigenetic mechanisms, this link remains inferred rather than directly demonstrated in patients. Clinical evidence in SLE specifically is especially scarce, being limited to observational and dietary intervention studies rather than randomized controlled trials. Given these constraints, the clinical findings summarized above should be regarded as preliminary signals supporting further investigation rather than as established evidence of disease-modifying efficacy.

## 5. Advanced Delivery Systems and Pharmacokinetics of Flavonoids

LUT, QUE, and AP are promising epigenetic and immune-modulating compounds. However, they dissolve poorly, are rapidly metabolized, and have low oral bioavailability. Because of this fact, it is difficult to reach sufficient concentrations of these particles in the plasma and tissues needed for meaningful epigenetic effects in vivo [25,107]. Achieving consistent, therapeutically relevant concentrations remains a major challenge [108,109]. Careful integration of mechanistic data from SLE and RA models with rigorous clinical study design would therefore be a sound strategy for turning nutraceutical research into formal autoimmune trials.

Conventional formulations, such as plant extracts or simple capsules, typically produce low and variable systemic exposure. This makes it uncertain whether the epigenetic effects observed in vitro can be translated into real clinical benefit. To address these limitations, a range of nanoformulations has been developed for QUE and related flavonoids [108,110,111]. This type of delivery system includes solid lipid nanoparticles, nanostructured lipid carriers, nanoemulsions, and protein-based nanocarriers [108,110,111]. These delivery systems are designed to protect flavonoids in the gastrointestinal tract and to improve their solubility and oral availability.

Nano-encapsulation can improve solubility, protect flavonoids from degradation in the gastrointestinal tract, and increase oral bioavailability. In some studies, peak plasma concentrations and overall exposure were increased up to two-fold compared with the free compound [107,112]. For example, QUE-loaded lipid nanocarriers and self-nanoemulsifying drug delivery systems show much better absorption without changing how quickly the compound is eliminated. This suggests that improved delivery, rather than a decreased metabolism, drives the higher exposure [113]. Several of these systems have also been designed to enhance delivery to inflamed joints in RA. These systems, which include intra-articular nanoemulsions and liposomes functionalized with ligands, recognize adhesion molecules or extracellular matrix components enriched in arthritic joints [114]. Also, QUE nanoemulsions incorporated into gels improved joint targeting and resulted in stronger reductions in TNF-α and synovial inflammation than non-encapsulated QUE, indicating that optimized delivery can translate into superior anti-inflammatory efficacy at the tissue level [25,115]. For SLE, kidney-targeted deliveries proved to be a promising way, since LN is driven by immune complex deposition and local immune activation in the glomerulus and interstitium. Thus, it is proposed that experimental nanocarriers that exploit particle size, surface charge, or ligand-based recognition of glomerular and tubular structures can concentrate anti-inflammatory or epigenetic agents in nephritic kidneys [116]. Adapting such functionalized liposomes or lipid nanoparticles for LUT, QUE, or AP could increase drug exposure at the renal sites, where DNMT1, METTL3, and other epigenetic regulators are dysregulated, while minimizing a systemic toxicity.

Across these delivery strategies, translation will hinge on linking drug exposure to biological effect by measuring plasma, and, where possible, tissue levels of nanoformulated flavonoids alongside epigenetic readouts such as DNMT1 or HDAC activity and DNA or histone modifications, allowing exposure–response relationships to guide dose selection and determine whether nanoformulations truly reach epigenetically effective concentrations in vivo [10,25].

A central translational limitation is that many of the in vitro studies discussed above used concentrations of flavonoids in a range of 20–100 μM or higher, whereas conventional oral supplementation of unformulated QUE, LUT, and AP was unlikely to achieve or sustain comparable systemic exposure because these compounds have poor oral bioavailability and rapid metabolism [5,55,75,108]. Accordingly, the epigenetic effects summarized in this review should be regarded as evidence of biological plausibility rather than as established in vivo therapeutic mechanisms, until pharmacokinetic–pharmacodynamic studies demonstrate target engagement at clinically achievable concentrations in relevant tissues.

## 6. Safety, Drug Interactions, and Regulatory Considerations

Although LUT, QUE, and API are widely described as natural or nutraceutical compounds, natural origin does not by itself establish safety, efficacy, or interchangeability with pharmaceutical-grade agents [117,118]. The available evidence suggests that these flavonoids are generally well tolerated in preclinical studies and in some short-term human interventions, but the safety database in SLE and RA remains limited, particularly for long-term use, higher doses, and co-administration with immunosuppressive therapy [118,119]. This limitation is important because the biological effects of flavonoids are often dose-dependent, and exposures that are insufficient for disease control may still be sufficient to alter metabolism or signalling pathways [119].

A further concern is their potential interactions with drugs. LUT, QUE, and API can modulate hepatic drug-metabolizing enzymes, including cytochrome P450 isoforms and UDP-glucuronosyltransferases, which raises the possibility of pharmacokinetic interactions with medicines commonly used in SLE and RA, such as methotrexate, corticosteroids, nonsteroidal anti-inflammatory drugs (NSAID), and biologic agents [117,118]. Because many patients with autoimmune disease require combination therapy, even modest effects on absorption, metabolism, or clearance could alter efficacy or toxicity profiles [117]. Accordingly, future studies should include formal drug-interaction assessments, standardized adverse-event reporting, and, where possible, exposure measurements alongside clinical outcomes [117,119].

## 7. Limitations and Future Perspectives

Despite the promising mechanistic and preclinical evidence summarized in this review, several limitations currently constrain the interpretation and translational significance of the available data. First, the current results are based mainly on in vitro experiments and animal models, whereas completed clinical studies on SLE and RA are scarce. Among the three flavonoids discussed, QUE is the only compound that has been evaluated in randomized clinical studies specifically in RA, while equivalent clinical evidence for LUT and API in either RA or SLE is lacking. Even for QUE, the available studies are limited by a small size of the tested population group, short intervention periods, heterogeneous doses and formulations in various independent studies, and the absence of standardized epigenetic endpoints, which makes it difficult to determine whether the observed clinical benefits are directly mediated through DNMTs, HDACs, histone marks, or non-coding RNAs in patients.

A second major limitation is the heterogeneity of the mechanistic literature. The studies reviewed differ substantially in compound concentrations, duration of exposure, disease model, cell type, and measured endpoint, which limits direct comparison across reports. In addition, a considerable proportion of the mechanistic evidence for HDAC inhibition, DNMT modulation, chromatin remodelling, and promoter demethylation derives from non-autoimmune systems, particularly cancer cell lines, rather than RA FLSs, lupus T cells, B cells, monocytes, or patient-derived tissues. Although these studies provide useful biochemical proof of principle, they should not be interpreted as direct evidence of therapeutic efficacy in RA and SLE diseases. Related to this, some apparently contradictory findings, such as concurrent reports of DNMT restoration in autoimmune models and DNMT inhibition in cancer models, reflect context-dependent and locus-specific effects rather than a single uniform direction of action, but they also highlight the need for more precise disease-specific mechanistic validation.

A third limitation concerns pharmacokinetics and formulation. LUT, QUE, and AP, all are characterized by poor solubility in water, rapid metabolism, and limited oral bioavailability, making it uncertain whether the concentrations used in many in vitro studies can be achieved or sustained in vivo. This creates a substantial translational gap between experimental plausibility and clinically meaningful target engagement. Although nano-formulations, lipid carriers, nano-emulsions, and related delivery systems appear promising for improving stability, absorption, and tissue targeting, these approaches remain insufficiently standardized and have not yet been linked consistently to pharmacokinetic–pharmacodynamic evidence in SLE- or RA-specific settings. Therefore, the epigenetic effects reported in the cell cultures have to be regarded as biologically plausible mechanisms rather than established in vivo therapeutic actions.

Another important limitation is that the data concerning non-coding RNA remain fragmentary. While miRNA-related mechanisms, particularly for QUE, are increasingly supported by disease-relevant studies, direct evidence involving lncRNAs and circRNAs in SLE or RA is still scarce. At present, the literature data are mainly focused on miRNA-mediated modulation than on lncRNA- or circRNA-dependent mechanisms, and this imbalance restricts the performance of comprehensive analysis and the establishment of a complex role of ncRNA in these processes. In practical terms, this means that the ncRNA landscape described in this review is still evolving and likely incomplete, especially for APn and LUT in autoimmune-specific models.

Safety and regulatory considerations are also important points to note. Although these flavonoids are widely described as natural compounds and are generally well tolerated in preclinical studies and some short-term human interventions, natural origin does not itself establish pharmaceutical-grade safety, efficacy, or interchangeability with approved therapies. Long-term safety data in SLE and RA remain limited, particularly at higher doses and in combination with corticosteroids, methotrexate, NSAIDs, or biologic agents. In addition, the possibility of pharmacokinetic interactions through modulation of the activity of the cytochrome P450 enzymes and other enzyme-related various metabolic pathways remains insufficiently studied in autoimmune populations that receive multidrug regimens.

Taken together, these limitations point to several priorities for future research. First, disease-specific mechanistic studies should increasingly rely on patient-derived immune cells, RA-FLSs, lupus nephritis-relevant renal cells, and well-characterized in vivo autoimmune models rather than extrapolation from unrelated systems. Second, future clinical trials should be on a larger scale, longer period, and better standardized with respect to a dose, formulation, and background therapy, and they have to incorporate direct epigenetic readouts such as DNMT and HDAC activity, locus-specific DNA methylation, histone acetylation patterns, and ncRNA profiling alongside clinical endpoints. Third, pharmacokinetic studies should be integrated with the development of formulation so that exposure–response relationships can be defined and epigenetically effective tissue concentrations can be established. Finally, biomarker-guided trial designs may be especially valuable. Baseline methylation abnormalities, miRNA signatures, cytokine profiles, and disease-specific epigenetic alterations could help identify patient subgroups most likely to benefit and move the field toward a more precise, mechanism-informed use of flavonoids as adjunctive therapies in SLE and RA.

## 8. Conclusions

Taken together, LUT, QUE, and AP emerge as safe, multi-targeted, natural epigenetic compounds that modulate DNMTs, HDACs, histone acetylation, and miRNA networks in ways that silence pathogenic Th1/Th17 responses, reduce autoantibody production, and restore regulatory pathways in SLE and RA models. Their broad anti-inflammatory, antioxidant, and epigenetic actions position them as attractive adjuncts or scaffolds for next-generation epigenetically informed therapies, provided that issues of bioavailability, standardization, and rigorous clinical testing are addressed. While studies are ongoing, researchers might consider combining flavonoids with conventional DMARDs or biologics as a rational strategy. By addressing the epigenetic and inflammatory baseline as seen in this review, flavonoids could reduce the overall inflammatory burden, thereby potentially enabling effective disease control with lower doses of methotrexate or anti-TNF agents and perhaps, improve response in partial responders.

The use of epigenetic biomarkers to monitor and individualize flavonoid therapy in SLE and RA could be another interesting key translational opportunity. Altered promoter methylation at CD11A, CD70, IL-17A, IL-2 and FOXP3, global DNA methylation changes in PBMCs, and dysregulated miRNA profiles, particularly miR-146a, and miR-21, among others, are characteristic anomalies in these diseases. Since LUT, QUE and AP have been shown to modulate DNMTs, HDACs, histone acetylation and these same miRNAs in preclinical studies, future clinical trials could incorporate baseline and on-treatment measurements of DNA methylation and miRNA signatures together with cytokines (IL-6, TNF-α, IL-17) and autoantibodies. The idea would be to confirm target engagement and to define epigenetic responders. If for an individual patient a particular flavonoid shows changes in methylation and miRNA patterns toward a profile corresponding to its healthier state, and parallel improvement in clinical and serologic measures, then that patient can be classified as an epigenetic responder. Over time, pooled trial data might reveal which baseline epigenetic signatures predict good response; for example, patients with marked CD70 hypomethylation and high miR-21 might benefit most from LUT or QUE. This would allow clinicians to select and adjust flavonoid therapy based on an individual’s epigenetic profile, rather than giving the same regimen to everyone.

## Figures and Tables

**Table 1:** Epigenetic potential of LUT, QUE, and AP in a relevant SLE model.

Flavonoid/ Study	Model/System	Epigenetic Pathway	Mechanism of Action	Results	Sources
**LUT/** **In vitro**	HeLa cell nuclear extracts; human/murine cell lines	DNMT1/MAPK-ERK	Dose-dependent DNMT inhibition; downregulates MAPK/ERK pathway to elevate DNMT1; reduces miR-21/miR-148a	↓ DNMT activity (35–66% at 5–20 μM); corrects hypomethylation at CD70/CD11A promoters; ↑ DNMT1 expression	[45,49,50]
**LUT/** **In vitro**	Human/murine immune & non-immune cell lines	HDAC1/2/7/10/11	Direct HDAC inhibition (HDAC1/2/7/10/11); ↓ HAT/HMT (H3K9) activity	↓ HDAC activity (28–55% at 5–20 μM); ↑ H3K9ac/H3K18ac on H3/H4; chromatin relaxation at immune-regulatory genes	[24,50]
**LUT/** **In vivo**	Collagen-induced arthritis (CIA) mice; PEA+LUT (1 mg/kg i.p., 10 days)	HAT/HDAC balance	HDAC inhibition; restores HAT/HDAC balance via ChIP	↑ Histone acetylation at anti-inflammatory promoters in splenic/peripheral immune cells	[51,52]
**LUT/** **In vivo**	MRL/lpr & NZB/W F1 lupus-prone mice (25–100 mg/kg daily)	DNMT1/ERK-MAPK; Dlk1-Dio3 miRNAs	↑ DNMT1 via ERK/MAPK restoration; reinstates Dlk1-Dio3 miRNAs; HAT/HDAC equilibrium	Normalized methylation at CD11A/CD70/IL-17A promoters; ↑ H3/H4 acetylation at pro-apoptotic/anti-inflammatory loci	[9,29,53]
**QUE/** **In vitro**	Human PBMCs, T cells, monocytes (5–100 μM, 24–72 h)	DNMT1/3A/3B	↓ DNMT1/3A/3B expression; corrects aberrant promoter methylation	↓ DNMT1/3A/3B mRNA/protein; normalized methylation at IL-2/FOXP3/p16 promoters	[45,54,55]
**QUE/** **In vitro**	HL-60 leukemia cells (25–100 μM, 6–12 h)	ERK/JNK-MAPK; HAT	HAT activation via ERK/JNK-MAPK; weak HDAC inhibition	↑ H3 acetylation at FasL promoter (75–100 μM)	[56]
**QUE/** **In silico**	Computational docking (Auto Dock) on DNMT1/HDAC1/2	DNMT1/ HDAC1/2 catalytic sites	Direct binding to catalytic sites (hydrophobic/H-bonds)	Predicted inhibition of DNMT1/HDAC1/2	[45,57]
**QUE/** **In vivo**	MRL/lpr, NZB/W F1 lupus mice & CIA (25–100 mg/kg/day, 2–12 weeks)	DNMT1/SIRT1; miR-146a	Reinstates DNMT1; ↑ SIRT1; normalizes miR-146a↑/miR-21↓	Normalized methylation at CD70/IL-17A; ↑ histone acetylation at anti-inflammatory loci	[58,59]
**QUE/** **Clinical**	RA patients (polyphenol-rich/QUE 25–100 mg/day, 8–12 weeks)	HDAC1/2/11; DNMT1; miR-146a	↓ HDAC1/2/11 & DNMT1 ex vivo in PBMCs	Enhanced global DNA methylation; improved miRNA profiles (miR-146a↑)	[60,61,62]
**AP/** **In vitro**	Human RA-FLS (10–40 μM)	HDAC1/2 (class I)	Inhibits class I HDACs (HDAC1/2)	↑ Global & p21 promoter H3/H4 acetylation; ↓ HDAC1/2 expression	[23,32]
**AP/** **In vitro**	Mouse skin epidermal JB6 P+ cells	DNMT1/3a/3b; HDAC1-8; Nrf2	↓ DNMT1/3a/3b & HDAC1-8; CpG demethylation at Nrf2 promoter	Dose-dependent Nrf2 promoter demethylation; ↑ Nrf2 mRNA/protein	[63]
**AP/** **In vivo**	CFA rats & CIA mice	HDAC expression	Suppresses HDAC expression in immune/joint tissues	↑ Global/promoter-specific H3/H4 acetylation in target tissues	[32,64,65]
**AP/** **In vitro**	SNF1 splenocytes (5–30 μM+nucleosome); lupus-prone Th1/Th17/B/DC cells	NF-κB/Th17; HDAC	HDAC inhibition; reduced accessibility at inflammatory promoters	Altered H3/H4 acetylation; chromatin tightening at NF-κB/Th17 loci	[23,33]
**AP/** **In vivo**	SNF1 (NZB×SWR F1) & MRL/lpr lupus mice	NF-κB/STAT3; HDAC/DNMT	Indirect HDAC/DNMT reduction; ↓ activating acetylation at STAT3 targets	Reduced chromatin accessibility at NF-κB/STAT3 promoters	[33,66]

↑ means an increase (up-regulation) of the process, ↓ means a decrease (down-regulation) of the process.

**Table 2:** MicroRNA, lncRNA, and circRNA evidence for LUT, QUE, and AP in SLE and RA models.

Flavonoid	ncRNA	Target/ Molecule	Mechanism	Type of Action/Evidence	Sources
**LUT**	miRNA	Dlk1-Dio3 cluster	Expression of this epigenetically silenced miRNA cluster reinstated	Lupus nephritis, MRL/lpr mice (direct action), in vivo	[29,86]
**LUT**	miRNA	miR-21, miR-148a	Proposed reduction, associated with restored DNMT1 expression	Mechanistic/inferred—based on the general role of these miRNAs in SLE hypomethylation; not directly measured in LUT-treated cells	[39]
**QUE**	miRNA	miR-146a ↑; GATA6 ↓	Increased miR-146a suppresses GATA6, reducing FLS migration/invasion	RA-FLS (direct action), in vitro	[90]
**QUE**	miRNA	miR-431-5p/MDM4	Regulated the miR-431-5p/MDM4 axis and promoted apoptosis of RA-FLS	RA-FLS (direct action), in vitro	[91]
**QUE**	miRNA	miR-146a ↑, miR-21 ↓, miR-27a ↓	Anti-inflammatory miRNA upregulated; pro-inflammatory miRNAs downregulated	Lupus-prone MRL/lpr mice, senescent CD4+ T cells, human PBMCs (direct action)	[59,88]
**QUE**	lncRNA	XIST ↓; miR-485 de-repressed; PSMB8 ↓	Suppressed XIST expression, de-repressing miR-485 to downregulate PSMB8 and decreasing inflammatory cytokine production	Human RA patient-derived FLS (direct action), in vitro	[92]

↑ means an increase (up-regulation) of the process, ↓ means a decrease (down-regulation) of the process.

## Data Availability

No new data were created or analyzed in this study. Data sharing is not applicable to this article.

## Abbreviations

H	Histone
DNA	Deoxyribonucleic acid
miRNA	microRNA
SLE	Systemic lupus erythematosus
DNMT	DNA methyltransferase
HDAC	Histone deacetylase
RA	Rheumatoid arthritis
FLSs	Fibroblast-like synoviocytes
LFA-1	Lymphocyte function-associated antigen 1
NSAIDs	Non-steroidal anti-inflammatory drugs
DMARDs	disease-modifying anti-rheumatic drugs
TNF-α	Tumour necrosis factor α
AP	Apigenin
LUT	Luteolin
QUE	Quercetin
NF-κB	Nuclear Factor kappa-light-chain-enhancer of activated B cells
IL	Interleukin
SIRT1	Silent Information Regulator 1
EZH2	Enhancer of zeste homologue 2
HMT	Histone methyltransferase
SAM	S-adenosylmethionine
Nrf2	nuclear factor erythroid 2-related factor 2
HO-1	Heme oxygenase 1
NQO1	NAD(P)H: Quinone Oxidoreductase 1
SOD	Superoxide dismutase
ROS	Reactive oxygen species
MS	Methionine synthase
CBS	Cystathionine β-synthase
MAPK	Mitogen-activated protein kinase
PI3K	Phosphatidylinositol 3-kinase
Akt	Protein kinase B
HAT	Histone acetyltransferase
ERK	Extracellular signal-regulated kinase
JNK	c-Jun N-terminal kinase
AP-1	Activator protein-1
CBP	CREB-binding protein
PEA	Palmitoylethanolamide
CIA	Collagen-induced arthritis
ChIP	Chromatin immunoprecipitation
NFATC2	Nuclear factor of activated T cells 2
PRKCE	Protein kinase C Epsilon
STAT3	Signal transducer and activator of transcription 3
LDHA	Lactate dehydrogenase A
H3K9la	histone H3 lysine 9 lactylation
MRL/lpr	Murphy Roths Large / lymphoproliferation strain
NZB/NZW F1	New Zealand Black/New Zealand White first filial generation hybrid strain
LN	Lupus nephritis
PBMCs	Peripheral blood mononuclear cells
FOXP3	Forkhead box P3
CRP	C-reactive protein
FLS	Fibroblast-like synoviocytes
CFA	Complete Freund’s Adjuvant
ANA	Antinuclear antibodies
CTL	Cytotoxic T lymphocytes
DAS	Disease activity score
ESR	Erythrocyte sedimentation rate
CSR	Class-switch recombination
CAT	Catalase
